# Endometrial Angiogenesis of Abnormal Uterine Bleeding and Infertility in Patients with Uterine Fibroids—A Systematic Review

**DOI:** 10.3390/ijms24087011

**Published:** 2023-04-10

**Authors:** Emma E. Don, Mei-An Middelkoop, Wouter J. K. Hehenkamp, Velja Mijatovic, Arjan W. Griffioen, Judith A. F. Huirne

**Affiliations:** 1 Department of Obstetrics and Gynecology, Amsterdam UMC, Location Vrije Universiteit Amsterdam, De Boelelaan 1117, 1081 HV Amsterdam, The Netherlands; 2 Amsterdam Reproduction and Development, De Boelelaan 1117, 1081 HV Amsterdam, The Netherlands; 3 Angiogenesis Laboratory, Department of Medical Oncology, Amsterdam UMC, Cancer Center Amsterdam, 1081 HV Amsterdam, The Netherlands

**Keywords:** abnormal uterine bleeding, angiogenic proteins, endometrium, infertility, female, leiomyoma, menorrhagia, neovascularization, pathologic, neovascularization, physiologic

## Abstract

Uterine fibroids are the most common benign tumors in women, with abnormal uterine bleeding (AUB) as the main reported symptom. Additionally, an association between fibroids and infertility has been established, especially if the fibroid protrudes in the uterine cavity. Hormonal therapy is associated with side-effects and as well as hysterectomy, which is incompatible with a desire to conceive. To improve treatment, it is essential to unravel the etiology of fibroid-related symptoms. We aim to evaluate endometrial angiogenesis in women with fibroids, with and without AUB, and the influence of pharmaceutical therapies in these patients. Furthermore, we explore the possible role of altered angiogenesis in patients with fibroids and infertility. We performed a systematic review according to PRISMA-guidelines (PROSPERO: CRD42020169061), and included 15 eligible studies. Endometrial expression of vascular endothelial growth factor (VEGF) and adrenomedullin was increased in patients with fibroids. This suggests aberrant angiogenesis, potentially involving disturbed vessel maturation, resulting in immature and fragile vessels. Treatment with gonadotropin-releasing hormone agonist, ulipristal acetate, and continuous oral contraception pills reduced several angiogenic parameters, including VEGF. If infertile and fertile patients with fibroids were compared, a significant decreased expression of the bone morphogenetic protein/Smad-protein pathway was found, possibly caused by the increased expression of transforming growth factor-beta. For future therapeutic development, these different angiogenic pathways could be of interest as possible targets to treat fibroid-related symptoms.

## 1. Introduction

Uterine fibroids or leiomyomas are the most common benign pelvic tumors in women and are a major cause of morbidity. Fibroids are heterogeneous in size, number, location, and vascularity and as a consequence, in symptomatology [1,2]. Symptoms are clinically present in about 25–50% of women with fibroids and include, for example, abnormal uterine bleeding (AUB), pelvic pressure, pain, and infertility. Uterine fibroids are the primary indication for hysterectomy worldwide [3,4] and are a major public health burden with annual costs of 30 million EUR in the Netherlands and five billion USD in the United States [5]. Although it is known that fibroids can cause a variety of symptoms, there is still a large knowledge gap that links the pathophysiology of fibroids to their clinical presentation. As hormonal therapy and hysterectomy are sometimes undesired in patients, there is an urgent need for alternative treatments that address the etiology of fibroid-related symptoms and improve them.

AUB that is caused by fibroids is classified by the International Federation of Gynecology and Obstetrics (FIGO) as AUB-L (AUB-Leiomyoma), with a sub-classification system that ranges FIGO types 0–8, based on their location within the myometrium [6,7]. According to FIGO, the diagnosis of AUB is present when the patient has an abnormal menstruation in terms of volume, duration, frequency, and/or the presence of an irregular cycle for at least six months [7]. Additional terminology is used for heavy menstrual bleeding (HMB), which is defined as excessive menstrual blood loss that interferes with a woman’s physical, social, emotional, and/or material quality of life [7,8,9].

Recent studies demonstrate the influence of fibroids on the endometrium. They showed that mechanotransductional changes can result in increased myometrial rigidity and decreased myometrial contractility, affecting endometrial bleeding, receptivity, and implantation. It was also shown that fibroids can regulate the transforming growth factor-beta (TGF-β) pathway and the expression of the extracellular matrix (ECM) production regulators, influencing the endometrial vascular system. In addition, imbalance in numerous other factors such as cytokines, hormones, and growth factors can influence and dysregulate endometrial growth and bleeding [10,11]. Moreover, this impaired or dysfunctional endometrium is thought to influence fertility in patients with fibroids, either by impaired endometrial receptivity, deviant local hormonal concentrations, chronic endometrial inflammation, and/or abnormal vascularization [12,13].

### Angiogenesis

**Figure 1:**
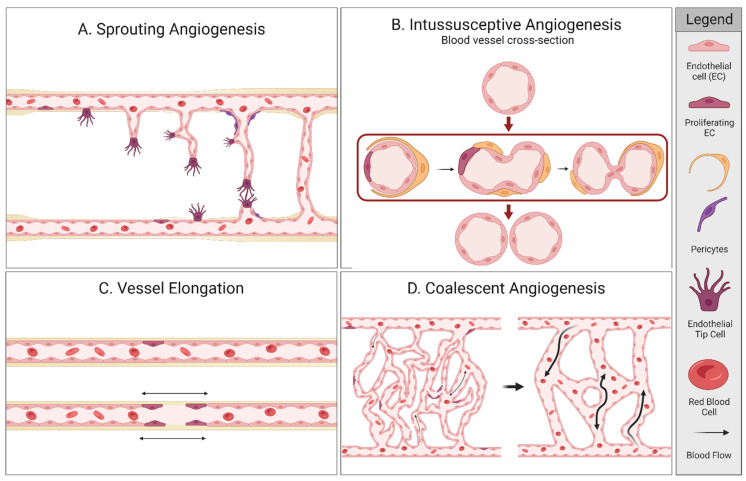
Different types of angiogenesis. Created with BioRender.com.

Angiogenesis is the process of forming new blood vessels out of pre-existing capillaries, driven by the requirement of oxygen [14]. There are several forms of angiogenesis as shown in Figure 1 and its accompanying Table 1. New blood vessels can be generated by; (1) sprouting angiogenesis, (2) intussusceptive angiogenesis, (3) vessel elongation, and (4) coalescent angiogenesis [15,16,17,18]. In sprouting angiogenesis, vessels grow from existing vessels by initiation of specialized endothelial cells (ECs), known as tip cells. With intussusceptive angiogenesis, the vessel splits into two daughter vessels [15,16,18,19]. Vessel elongation (or remodeling) involves passive cell growth as the vessel widens or extends by proliferating ECs [17,20]. Recently, a fourth form of angiogenesis has been introduced, coalescent angiogenesis. Coalescent angiogenesis is described as the process of the fusion (or coalescence) of an isotropic capillary mesh into a new extended vessel by parallel processes of vessel maturation and regression. In contrast to sprouting, coalescent angiogenesis can result in fast extension of the primary vessels while preserving perfusion. In addition, it seems that this form of angiogenesis does not need hypoxic triggers, can increase blood flow, and may even result in a decreased or unchanged capillary density [18,21]. A separate angiogenic mechanism is mediated through the embodiment of circulating endothelial progenitor cells in the existing vessel wall, contributing to sprouting angiogenesis, intussusception, and elongation [17,22].

Normal endometrial growth during the proliferative phase of the menstrual cycle is required for successful embryonic implantation and necessitates a well-regulated angiogenic process. In the endometrium, this process is driven by angiogenic factors, influenced through hypoxia and ovarian steroid hormones [23,24]. Recently, a systematic review studied aberrant angiogenesis in several types of AUB as a significant point of interest for the development of anti-angiogenic therapy in patients with AUB. This review reported that aberrant angiogenesis in endometrial and iatrogenic AUB (AUB-E and AUB-I, respectively) showed impaired vessel maturation, leading to fragile vessels and changes in vessel density [25]. A second review investigated angiogenesis in infertility and adenomyosis-associated AUB (AUB-A) and reported similar results [26]. A third review reported on the impact of fibroids on the human endometrium and showed that disturbed angiogenesis is one of the key factors in explaining AUB in an abnormal endometrium. Nonetheless, a schematic and complete overview of the regulating mechanisms in patients with fibroids or AUB-L is still missing [10]. This current systematic review of the literature investigates the role of angiogenesis in the endometrium of patients with fibroids compared to patients without fibroids. We will focus on patients with fibroids, with or without AUB and/or the use of exogenous pharmaceutical therapies. The possible role of aberrant angiogenesis on fertility problems in these patients will also be explored. 

## 2. Materials and Methods

### 2.1. Protocol Registration

This systematic review was initially designed to cover aberrant angiogenesis in the etiology of AUB and infertility in patients with and without fibroids. As the results were too extensive for one single review, it was decided to create two separate systematic reviews with two systematic literature searches. The initial protocol is registered in the PROSPERO database in July 2020 (CRD42020169061) and conducted according to the PRISMA guidelines (Appendix 1) [27]. The first systematic review, showing the results of aberrant angiogenesis in AUB-E and AUB-I in patients without fibroids, is published previously [25]. 

### 2.2. Data Source and Search Strategy

For this systematic review, two systematic literature searches were performed in collaboration with a medical information specialist and updated until 16 June 2022 in the following databases: PubMed, Cochrane Library, Embase, and Web of Science. Search I comprised of angiogenic-related outcomes in patients with fibroids with and without AUB, and Search II consisted of angiogenic-related outcomes in patients with fibroids and infertility. The following search terms and their synonyms were used in Search I: ‘fibroids’, ‘AUB’, ‘angiogenesis’ and several pro- and anti-angiogenic parameters. For Search II, ‘AUB’ was replaced for ‘infertility’ and its synonyms such as ‘subfertility’. The full search strategy and terms can be found in the Supplementary Information (Supplemental File S1). 

### 2.3. Eligibility Criteria and Study Selection

Both searches included original research papers only. This included randomized control trials (RCTs), cohort studies, and case-control studies that focused on an association between angiogenesis in the endometrium of patients with fibroids. No language restrictions or limitations regarding publication year were applied. Papers that assessed the association of angiogenesis in the endometrium in patients with exogenous hormones were included and reported separately. The following articles were excluded: meta-analyses, case-reports, letters, reviews or editorial articles, and studies on in vitro cell cultures that did not study patients with AUB or fibroids. Papers that studied angiogenic factors in menstrual effluent or serum levels without biopsies of the endometrium, were also excluded (See Table 2). Two authors (ED and MM) independently screened titles and abstracts. If eligibility was expected, the full article was acquired and reviewed. Any disagreements were resolved by discussion. Additionally, the references were checked for remaining eligible studies. 

### 2.4. Data Extraction and Quality Assessment

Data from the published studies were extracted independently by the authors (ED and MM) using a standardized form that included several characteristics such as study design, the number of patients, and outcomes. The included studies reported on patients with uterine fibroids and were divided in four groups: (1) fibroids versus no fibroids, (2) fibroids with AUB versus no fibroids and AUB, (3) fibroids with, versus without exogenous hormones, and (4) fibroids and infertility. The results were subdivided in outcomes of angiogenesis-related vessel morphology and angiogenic parameters, including growth factors and their receptors.

Two authors (ED and MM) independently performed a quality assessment of the included studies by using the Cochrane Risk of Bias tool version 2.0 (RoB 2) for RCTs and the Newcastle–Ottawa Scale (NOS-RoB) for cohort studies (Supplemental File S2) [28,29,30,31]. To assess comparability in the NOS-RoB, two population characteristics were chosen as important potential confounders: history of hormonal therapy use [32] and if patients were of reproductive age, whereas reproductive age ranged from 15 to 49 years, as defined by the World Health Organization (WHO).

### 2.5. Study Selection

**Figure 2:**
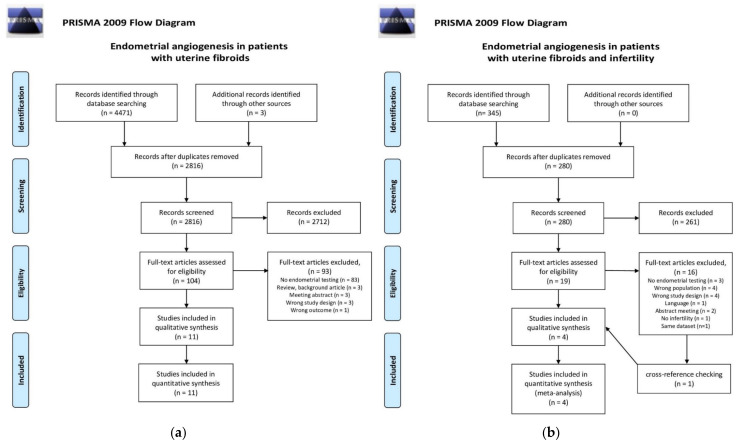
PRISMA 2009 flow diagram for the two searches that were carried out for this systematic review [33]. (**a**) Search I: Endometrial angiogenesis in patients with uterine fibroids; (**b**) Search II: Endometrial angiogenesis in patients with uterine fibroids and infertility.

The first search that assessed AUB resulted in 4471 references. After removal of duplicates and title-abstract screening, 104 full-text articles were assessed for eligibility. A total of six additional articles were included after the cross-reference check. In total, 11 articles were included in this review, as shown in Figure 2a. The second search that assessed infertility resulted in 345 articles, of which 19 were considered eligible for full article screening. After cross-reference checking, a total of four articles were included. The search flow diagram is shown in Figure 2b.

## 3. Results

### 3.1. Study Characteristics

The study characteristics of the included studies are presented in Table 3. Study characteristics were presented in four subgroups, as explained in the methods section. Patient populations were considered to be matching for reproductive age and history of hormonal therapy use in accordance with the RoB-assessment. AUB was not described in four studies [34,35,36,37] and was subjectively defined in five studies, based on patients’ medical history [38,39,40,41,42].

The included studies based the menstrual cycle phases on (1) histology according to the Noyes criteria or Fox and Buckley criteria, (2) patients medical history or cycle day, or (3) this was not specifically described [43,44]. The studies defined up to seven phases of the menstrual cycle: menstrual, early–mid–late proliferative, and early–mid–late secretory phases. This review reports the results for two phases; the proliferative and secretory phase. For the results regarding exogenous hormone supplementation, phases were not defined as endometrium that is exposed to hormones does not show different histological phases. Hormone type and length of use is specified, as shown in Table 3. The presented outcomes are defined as vascular morphology outcomes or as angiogenic parameters and include when studied, both proteins and receptors.

**Table 3:** Study characteristics of included studies.

Patients with Uterine Fibroids (UF), Divided in Four Study Groups									
Study, Year	Study Type	Total N	Study Group	Control Group	Matching ^a^	Menstrual Cycle Phase ^b^	Methods ^c^	AUB Classification	
***1. Uterine Fibroids***			***UF***		***No UF***				
Bereza et al. 2014 [45]	Retrospective case-control	22	15	7	No	7 phases	CCT	NA	
Hague et al. 2000 [38]	Case-control	91	52	39	Yes	3 phases	IHC	Subjective complaint of HMB in control group	
Governini et al. 2021 [46]	Case-control	36	18 (FIGO type 3)	18	Yes	1 phase (proliferative)	IF; RT-PCR; WB	NA	
***2. AUB and UF***			***UF and AUB***		***No UF/AUB***				
Anania et al. 1997 [39]	Case-control	28	14	14	Yes	3 phases	IHC; RT-PCR; WB	Subjective complaint of HMB	
Makhija et al. 2008 [34]	Case-control	118	UF: 55	63	No	5 phases	IHC	Not described	
Oh et al. 2013 [35]	Case-control	32	UF: 24 Sympt.: 17 Asympt.: 7	8	Yes	2 phases	IHC	Not described	
Zhang et al. 2010 [40]	Case-control	90	UF: 60 Sympt.: 29 Asympt.: 31	30	Yes	2 phases	IHC	Subjective complaint of AUB	
***3. Patients with uterine fibroids with and without pharmaceutical therapy***									
**Study, year**	**Study type**	**Total N**	**Study group with therapy**	**Control group**	**Matching ^a^**	**AUB at start of study (N) ^d^**	**Methods ^c^**	**AUB classification**	**Exogenic hormone ^e^** **(length of use)**
Maia et al. 2008 [36]	Retrospective cohort	118	40	78	NA	*Study*: AUB (9), no MBL(31) *Control*: HMB + UF	IHC	Not described (indication: hysterectomy)	Continuous Gestinol (1–24 months)
Khan et al. 2010a [42]	Prospective case-control	56	20	36	No	*Study*: HMB *Control*: HMB	IHC, ELISA	Subjective complaint of AUB	GnRHa (3–6 months)
Khan et al. 2010b [41]	Prospective case-control	56	20	36	No	*Study*: HMB *Control*: HMB	IHC, BrdU-assay	Subjective complaint of AUB	GnRHa (3–6 months)
Kolanska et al. 2019 [37]	Retrospective case-control	45	14	27	No	Not described	IHC	Not described (indication: hysterectomy)	UPA (3 months)
***4. Patients with uterine fibroids and infertility***									
**Study, year**	**Study type**	**Total N**	**Study group**	**Control group**	**Matching ^a^**	**Methods ^c^**	**Patient sample selection ^f^**		
Doherty et al. 2015 [47]	Experimental in vitro study	14	10	4	No	ESCs + FCM exposure; RT-PCR	**UF**: LCM cultured from patients after myomectomy or hysterectomy. **C**: ES from fertile controls (no hormonal contraception or uterine pathology)		
Kozachenko et al. 2020 [48]	Prospective case-control	20	10	10	No	IHC	**UF**: IVF or ICSI indication, ES before and after surgery **C**: IVF or ICSI indication because of tuboperitoneal infertility factor without endometrial or myometrial pathology		
Novin et al. 2018 [49]	Prospective case-control	20	10	10	No	IHC	**UF**: infertile patients, which after myomectomy reported a successful pregnancy. **C**: at least one natural labour, no hormonal contraception or uterine pathology and a normal menstrual cycle		
Sinclair et al. 2011 [50]	Prospective case-control and in vitro study	24	12	12	No	ESC culture; RT-PCR; IHC; ELISA	**UF**: Endometrial samples after myomectomy for fertility (8) or hysterectomy (4). **C**: ES after undergoing laparoscopic (7) or hysteroscopic tubal sterilization (5); without evidence of uterine pathology		

^a^ Matching for age and hormonal use, NA: not applicable, ^b^ 2 phases: proliferative (P) and secretory (S); 3 phases: menstrual (M), P, and S; 4 phases: P, early (ES), mid (MS), and late secretory (LS); 5 phases: M, P, ES, MS, and LS; 6 phases: M, early proliferative (EP), mid proliferative (MP), ES, MS, and LS; 7 phases: M, EP, MP, late proliferative (LP); ES, MS, and LS, ^c^ BrdU-assay: 5-bromo-2-deoxyuridine incorporation assay; CCT: Corrosion casting technique; ELISA: enzyme-linked immunosorbent assay; ESCs + FCM exposure: cultured endometrium cells (from patients without fibroids) were exposed fibroid-conditioned media; IF: Immunofluorescence; IHC: immunohistochemistry; RT-PCR: Real-time polymerase chain reaction; WB: Western-Blot, ^d^ AUB: abnormal uterine bleeding; HMB: heavy menstrual bleeding or menorrhagia; no MBL: no menstrual bleeding, defined as amenorrhea, no bleeding/spotting days during the reference period; NMB: eumenorrhea, normal blood loss; UF: uterine fibroids, ^e^ Gestinol: 75 mcg gestodene + 30 mcg ethinylestradiol; GnRHa: gonadotropin-releasing hormone agonist; UPA: Ulipristal acetate, ^f^ C: control group; ES: endometrial sampling; ICSI: intracytoplasmic sperm injection; IVF: in vitro fertilization; LCM: Leiomyoma-conditioned media; UF: uterine fibroids.

### 3.2. Assessment of Risk of Bias

Search I included 10 case-control studies and one cohort study, and Search II included four case-control studies. The RoB-assessment of all the included studies is shown in the Supplementary File S3. The cohort study was of ‘good’ quality, according to the NOS. Of the 15 case-control studies of both Search I and II, four were of ‘good’, five of ‘fair’, and five of ‘poor’ quality. In general, the quality was defined as ‘fair’ when the non-response rate was not described and if some steps in the selection process of the included studies were poorly reported. If groups were not comparable or if more steps of the study selection and/or data to assess the comparability of the included groups were missing, this resulted in a ‘poor’ quality assessment. However, the ascertainment of exposure, and methods of ascertainment were correctly performed overall in all the included studies.

### 3.3. Summary of Several Angiogenic Pathways

**Figure 3:**
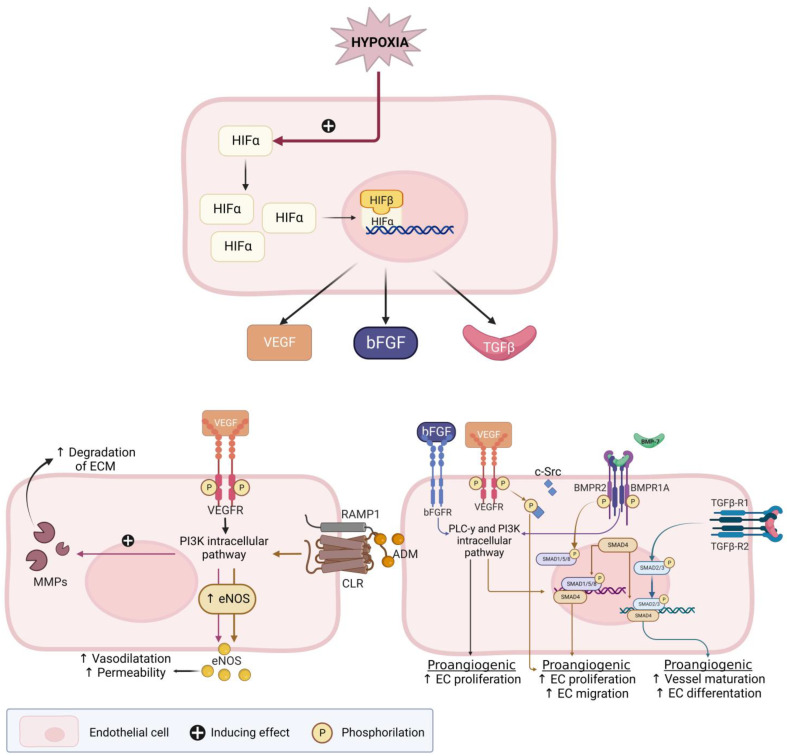
Hypoxia-inducible factor (HIF)-pathway and its effect on several pro-angiogenic factors. Adapted from a Middelkoop et al. 2023—Created with BioRender.com [25]. Figure legend: ↑ (upward arrow): indicates an increased process; e.g., an increased vasodilatation; ADM: adrenomedullin; bFGF: basic fibroblast growth factor; BMP(R): bone morphogenetic protein (receptor); CLR: calcitonin receptor-like receptor; c-Src: cellular Src; EC: endothelial cell; ECM: extracellular matrix; eNOS: endothelial nitric oxide synthase; FGF-R: FGF-receptor; HIFα/β: Hypoxia inducible factor; MMP: matrix metalloproteinases; NO: nitric oxide; PLC-γ: Phosphoinositide phospholipase C—pathway; PI3K: phosphoinositide 3-kinase—pathway; RAMP: receptor-activity-modifying proteins; Smad: Suppressor of mothers against decapentaplegic; TGF-β(R)1/2: transforming growth factor-β (receptor) 1 or 2; VEGF(R): vascular endothelial growth factor (receptor).

A number of pro- and anti-angiogenic factors are discussed in this review and this section provides an overview of the reported factors and receptors which are displayed in Figure 3. In reaction to ischemic conditions or hypoxia, ECs have to migrate and proliferate to the area in need of oxygen. In order for ECs to enter the angiogenic cascade, vascular relaxation is necessary. The first discovered angiogenic factor was of the fibroblast growth factor (FGF) family. FGF-2, also known as bFGF, stimulates many important steps of the angiogenesis cascade. Additionally, interaction with other growth factors can accelerate EC function [51].

Endometrial nitric oxide synthase (eNOS) is the predominant isoform of NOS in the human endometrium and produces nitric oxide (NO) [52]. NO stimulates vasodilatation and EC permeability and NO production is stimulated by vascular endothelial growth factor (VEGF) and the binding of adrenomedullin (ADM) to the calcitonin receptor-like receptor (CLR) [53,54]. VEGF is a crucial activator of EC function and is involved in all steps of the angiogenesis cascade, including EC migration and proliferation. VEGF also increases the expression of matrix metalloproteinases (MMPs) and plasminogen activators that degrade ECM. The VEGF-family consists of six different types of growth factors, of which VEGF-A and the VEGF receptor 2 (VEGFR2) interaction plays a major role in sprouting angiogenesis [51]. Estrogen treatment increases VEGF, while a combination of estrogen and progesterone receptor antagonist treatment reverses the estrogen-stimulated VEGF, suggesting that progestins could reduce production of these angiogenic factors by binding to the progesterone receptor (PR) [55].

ADM is part of the calcitonin family and has been shown to be involved in endometrial angiogenesis and regulate endometrial angiogenesis by binding to the CLR. ADM is also a vasodilator and promotes angiogenesis by EC growth, stabilizing the endothelial barrier that surrounds the vessel. Therefore, a decrease in ADM can lead to an increased barrier permeability, resulting in vessel leakage and an increased blood loss [56,57,58,59]. The cyclooxygenase (COX)-2-prostaglandin pathway is also known to influence VEGF expression. The COX-enzyme was increased in patients with HMB, making this an interesting factor to assess in patients with fibroids [60].

**Figure 4:**
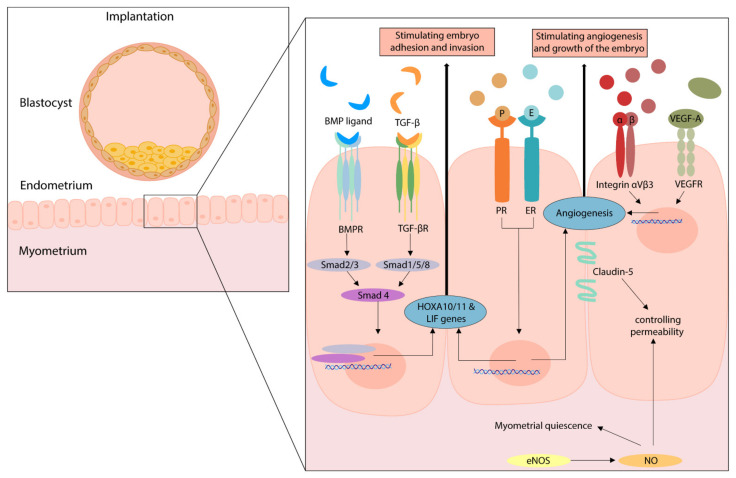
The effect of several angiogenic pathways on implantation. Figure legend: BMP(R): Bone morphogenetic protein (receptor); EC: endothelial cell; E(R): Estrogen (receptor); eNOS: endothelial nitric oxide synthase; LIF: leukemia inhibitory factor; NO: nitric oxide; P(R): progesterone (receptor); Smad: Suppressor of mothers against decapentaplegic; TGF-β: transforming growth factor; TGF-β(R): transforming growth factor-β (receptor); VEGF-A/R: vascular endothelial growth factor-A/receptor.

Another explored group of potentially involved angiogenic factors in the human endometrium is the bone morphogenetic protein (BMP) family, which is also displayed in Figure 4. They activate downstream ‘suppressor of mothers against decapentaplegic’ (Smad) proteins and function as regulatory factors in the TGF-β superfamily [61]. For example, TGF-β activates Smad2/3, which by several pathways mediates vessel maturation and EC differentiation [62]. Finally, it has been suggested that fibroids stimulate a chronic inflammatory environment where cytokines, next to fibroid pathogenesis and growth, are thought to be involved in the fibroid symptomatology and infertility. This inflammatory environment attracts cytokines such as interleukins (ILs [11,63], or ‘signal transducer and activator of transcription’ (STAT) genes. Both ILs and STAT genes are suggested to play a role in angiogenesis [11,46,63].

### 3.4. Summary of Angiogenic Pathways during Implantation

A number of angiogenic factors that are involved in the implantation process are discussed in this review and displayed in Figure 4. Next to its angiogenic function during the menstrual cycle, eNOS is an important regulator of myometrial quiescence during the gestational period. eNOS overexpression can induce cellular apoptosis and/or result in nitrosylation of key signaling proteins. Thus, increased eNOS expression may impair endometrial and myometrial function [49,64]. The overexpression of endometrial eNOS is described for endometriosis, adenomyosis, recurrent miscarriages, and unexplained infertility [65,66]. Since fibroids are an estrogen-, and progesterone-dependent pathology and endometrial eNOS is also regulated by these hormones, eNOS overexpression might be an important factor for patients with fibroids and infertility [49].

Furthermore, examples of key regulators genes of implantation are Homeobox-A10 and A11 (HOXA10/11) and leukemia inhibitory factor (LIF). If one of these genes is altered in mice, this results in infertility due to failed endometrial receptivity [47]. No human mutations are known in these genes, however, patients with conditions known for implantation defects such as endometriosis, hydrosalpinx, and polycystic ovary syndrome, have showed a diminished gene expression [47]. HOXA10 is also involved in the formation of integrin αVβ3, which was identified as a marker of the implantation window, and its localization and expression is proposed as a potential receptor for embryo adhesion [48]. Furthermore, integrin αVβ3 is an essential molecule that is involved in tumor angiogenesis [67,68]. In addition, alterations in the TGF-β or BMP/Smad pathway could lead to disturbed endometrial receptivity, since these pathways regulate HOXA10/11 and LIF expression [47,50]. Especially BMP-2 is critical during normal gestation, since it is shown to support implantation. Therefore, decreased levels of BMP-2 can lead to failed decidualization and the failure to support embryo implantation [69].

Finally, estradiol and progesterone regulate angiogenesis in diverse settings, also including the processes of implantation and gestation. They specifically stimulate the LIF pathway but are also involved in the regulation of other angiogenic factors, such as NOS, VEGF, bFGF, and hypoxia-inducible factor (HIF). Next to embryo adhesion and invasion, adequate endometrial vascularization is crucial for successful implantation. In human endometrium, VEGF-A is the main pro-angiogenic factor that stimulates this angiogenesis and by itself is stimulated by estradiol [70]. Additionally, while studies on tight junctions in the endometrium are limited, it is proposed that Claudin-5 could be used as a marker for endometrial receptivity [71].

### 3.5. Results of Individual Studies

Patients with and without fibroids—A total of three studies compared the endometrial tissue of patients with and without fibroids, of which the results are presented in Table 4. One study found a significant increase in venous lakes in the endometrium of patients with fibroids compared to patients without fibroids. These venous lakes are described as dilated veins and could be interpreted as increased vascular diameter or could hypothetically also be a phase of coalescence angiogenesis [45]. The micro vessel density (MVD) was investigated by a different study and was not significantly different between patients with and without uterine fibroids. This study also compared several angiogenic parameters with no differences in aFGF and bFGF. However, an increased VEGF and ADM expression was found in patients with uterine fibroids, suggesting increased angiogenesis in these patients [38]. However, both the studies of Hague et al. 2000 and Bereza et al. 2014 were classified as poor quality in our RoB analysis (Supplementary File S2). A third study showed a trend for Increased VEGF, COX1/2, and STAT3 levels in patients with FIGO type 3 fibroids. These specific fibroids make close contact with the endometrium and induce impression of the uterine cavity, but the differences were not statistically significant. They did report significantly increased IL-1β/6/10 levels in the endometrium of uterine fibroids that indirect may result in increased cell proliferation and angiogenesis due to increased inflammation [46].

Patients with fibroids and AUB—A total of four studies compared patients with uterine fibroids and AUB with control patients without fibroids and AUB and the results are shown in Table 4. One study found an increase of FGF-R1 in the secretory phase in patients with symptomatic uterine fibroids [39]. A second study that was classified as poor quality in our RoB analysis (Supplementary File S2) found no differences in MVD, vascular congestion, and vascular dilatation between patients with fibroids compared to normal controls [34]. VEGF and eNOS were found to be increased in both the proliferative and secretory phases in patients with uterine fibroids in two studies. This increase was even more present in both patients’ subgroup with symptomatic uterine fibroids [35,40].

### 3.6. Effects of Pharmaceutical Therapy in Patients with Fibroids

Our search identified four articles reporting on the effect of pharmaceutical therapy in patients with symptomatic fibroids, as shown in Table 3. The included studies involve fibroid patients with complaints of AUB or HMB, FIGO classified as AUB-L. These studies examined the effect of gonadotrophin-releasing hormone agonist (GnRHa), continuous treatment with oral contraceptive pills (OCP), and ulipristal acetate (UPA), the results of which are shown in Table 5. Contradictory angiogenic effects would be expected in endometrial samples of patients with AUB-L treated with exogenous hormones compared to non-treated patients with AUB-L.

In line with this hypothesis, one study found a significant decrease in von Willebrand factor (vWF)-positive MVD in fibroid samples after treatment with GnRHa, which indicates an angiogenic decreasing effect of this treatment. Monocyte chemotactic protein 1 (MCP-1) is a chemokine involved in the recruitment of monocytes and was found to be significantly decreased in the endometria of patients with AUB-L and GnRHa treatment. Additionally, CD68-positive macrophage infiltration is known to be involved in an inflammatory response and was significantly decreased in these patients. These outcomes were irrespective of the menstrual cycle phases of the control samples [42]. In addition, a different study found a significant decrease in epithelial cell proliferation and a dose-dependent effect on epithelial and stromal cell proliferation in GnRHa-treated endometrium of patients with AUB-L compared with controls [41].

Immunostaining for VEGF expression was dominantly observed in the stromal cells of fibroid-exposed endometrium samples. These samples showed a significant decrease of VEGF expression after continuous exposure of OCP (containing 75 mcg gestodene + 30 mcg ethinylestradiol) compared to both the proliferative and secretory phases of non-treated controls with AUB-L [36]. COX-2 is another important angiogenic mediator and was seen in endometrial glands and surface epithelium. Endometrial samples of OCP-induced amenorrheic patients with uterine fibroids showed a significant decrease in COX-2 expression compared to the untreated control samples in the proliferative phase and no significant difference in the secretory phase. COX-2 expression in endometrial samples of fibroid patients with break-through-bleeding (BTB) after continuous OCP exposure was less suppressed compared to the untreated controls, with no significant difference of COX-2 in the proliferative phase and an increased expression of COX-2 in the early and late secretory phases [36]. As VEGF is an estrogen-responsive angiogenic factor, some studies assessed different parameters in the estrogen pathway [55]. Aromatase is an important local estrogen production regulator and was found to be significantly reduced in samples after continuous OCP treatment compared to the proliferative and early luteal phase of control samples [36].

One study compared endometrial samples before and after UPA, although it was classified as poor quality in our RoB analysis (Supplementary File S2) [37]. A prominent marker for endometrial epithelium maturation is the estrogen receptor (ER), which was significantly increased after three months of UPA treatment compared to samples of the no-UPA secretory phase, except in the stromal compartment of the basal layer. PR is another prominent marker for endometrial epithelium maturation. Compared to the non-treated endometrium, PR was significantly increased in all layers of the UPA-exposed endometrium of patients with AUB-L with the exception of the stromal compartment [37]. Progesterone induces expression of VEGFR2, which interacts with VEGF-A. UPA treatment showed a mixed agonist–antagonist effect on VEGFR2 in both endometrial layers, although these results were not significant [37]. UPA treatment did not change cell proliferation in the basal layer of the endometrium, although changes were observed in the superficial layer. In the UPA-treated endometrium samples, epithelial and stromal cell proliferation was decreased compared to the proliferative phase endometrium of controls without UPA use. However, compared to the secretory phase endometrium samples of non-UPA controls, epithelial cell proliferation was increased and stromal cell proliferation was unchanged [37].

### 3.7. Angiogenesis in Relation to Fibroids and Infertility

The second search regarding angiogenesis in the endometrium of patients with fibroids in relation to infertility resulted in four eligible articles. The results of these studies are shown in Table 6 and the involved factors are displayed in Figure 4. It is important to note that all studies used different inclusion criteria for the study and control group, as seen in basic characteristics which are displayed in Table 3. The study of Novin et al. 2018 found an increased expression of eNOS in infertile patients with fibroids compared to fertile controls without fibroids [49]. The study of Kozachenko et al. 2020 compared the endometrium of infertile patients with fibroids to infertile patients without fibroids, although this was classified as poor quality in our RoB analysis. This study found decreased levels of HOXA11 and LIF in the endometrium of infertile patients with fibroids compared to the control samples. Other markers for endometrial receptivity, ER, PR, VEGF-A, integrin αVβ3, and Claudin-5 (CLDN-5) were significantly decreased in patients with fibroids and infertility [48]. BMP-2 secretion is essential in decidualization and Sinclair et al. 2011 presented that cultured endometrial stromal cells (ESC) of fibroid patients expressed significant lower levels of BMP-2 compared with the controls. Moreover, after recombinant BMP-2 treatment, control ECS showed an increased expression of HOXA10 and LIF genes, while treatment of fibroid ECS showed a significant reduction compared to the treated control samples. This could potentially be caused by the increased expression of TGF-ß3, which is produced in large amounts by fibroid cells because TGF-ß3 downregulates BMPR-2 [50]. Immunostained endometrium from patients with fibroids showed reduced expression of BMPR-1A, -1B, and -2. Moreover, TGF-ß3 treatment showed a significant reduction in BMPR-1A, 1B, and -2 gene expression in fibroid ECS [50]. Furthermore, Doherty and Taylor et al. 2015 demonstrated in an in vitro experiment that ECS showed significant decreased levels of BMPR-1A and -2 after exposure to fibroid cell conditioned media. This resulted in decreased levels of HOXA10 and LIF, all under the influence of TGF-ß3 [47].

## 4. Discussion

### 4.1. Main Findings

This systematic review provides a summary of the available literature on angiogenesis in the endometrium of patients with fibroids with or without AUB, the influence of pharmaceutical therapies in these patients, and the possible role of altered angiogenesis in patients with fibroids and infertility. The angiogenic parameters ADM, eNOS, and VEGF were increased in all the reported fibroid groups, with even higher outcomes of eNOS and VEGF in the AUB-L patients. This is in line with our previous review that showed increased eNOS and VEGF levels in patients with AUB-E compared to normal controls [25]. The MVD, vascular congestion and dilation were similar in patients with and without fibroids. However, patients with fibroids showed more venous lakes compared to patients without fibroids, which could indicate an increased vessel diameter in fibroid patients. A larger vessel diameter could suggest that these vessels are fragile and leaking as a result of impaired vessel maturation. Unfortunately, vessel maturation was not examined in the included studies. Neither did any study examine vascular morphology in the endometrium in relation to fibroids and fertility. The current results show that patients with fibroids have increased pro-angiogenic parameters in the endometrium, with a more distinct significant effect in patients with AUB-L, suggesting increased angiogenesis.

However, two studies showed no difference in MVD in patients with and without fibroids and the presence of complaints of AUB did not seem to affect these outcomes [34,38]. Nevertheless, it is important to emphasize that the MVD is not equivalent to the degree of angiogenic activity [72,73]. As discussed in the introduction, it is unknown which angiogenic mechanism is responsible for angiogenesis in the endometrium and different mechanisms can have contradictory effects on the MVD. For example, sprouting angiogenesis increases the MVD and coalescent angiogenesis reduces MVD, while both mechanisms increase blood flow [15,18]. Furthermore, as the MVD is a relative number, the results can be influenced by volume changes in other cell components such as stroma or glandular cells or by shifting of edema [34,73]. For example, if the extravascular component of the endometrium decreases under the influence of medical agents or decreased estradiol levels, this may lead to an unchanged or increase of the MVD, even when the amounts of vessels or their diameters increase. Therefore, subtle reductions in vascularity may remain undetected in case of (induced) atrophy.

One study that assessed the effect of continuous OCP (gestodene/ethinylestradiol), showed decreased levels of VEGF, aromatase and COX-2, suggesting an anti-angiogenic effect of OCPs. Moreover, in OCP-users with BTB, COX-2 levels were increased, compared to both controls with AUB-L and OCP-users with amenorrhea [36]. This supports the possible relationship between increased COX-2 levels and BTB. As COX-2 is also known to influence VEGF-levels, this outcome is in line with the findings of a previous review that showed increased VEGF-D levels after short time (<4 months) of progesterone exposure from a levonorgestrel-releasing intra-uterine system [74]. The latter therapy is known to cause BTB in the first six months after insertion.

One other study demonstrated no difference in the expression of VEGFR2 after UPA. However, epithelial and stromal cell proliferation was decreased in the proliferative phase [37]. Since ER and PR immunostaining was increased after UPA use, the anti-angiogenic effect of UPA which results in amenorrhea, seems mostly based on the progesterone receptor antagonist effect and not merely on changes in VEGF. However, the exact mechanism behind this is still unknown [37,75]. Two studies researched the effect of GnRHa in the endometrium of patients with fibroids and indicated an anti-angiogenic effect, as they showed a decrease in MVD, macrophage infiltration, MCP-1, and endothelial, epithelial, and stromal cell proliferation [41,42]. This is in line with a study that evaluated the vascular index (VI) using 3D power Doppler in women with fibroids who were treated with GnRHa or UPA. They reported a very strong decrease in VI after 3 months of GnRHa use and a strong reduction in fibroid volume and AUB-L. After 3 months of UPA this effect was less consistent, with some patients showing an increase in VI, associated with an increase in fibroid volume and no effect on AUB-L. A limited number of patients reported a decrease in VI and associated fibroid volume and AUB-L. As our review shows some interesting results of angiogenic parameters in patients with AUB-L after pharmaceutical therapy, more research is needed to elucidate UPA’s possible anti-angiogenic effect [76].

This review found only four eligible studies that researched angiogenic factors in the endometrium of patients with fibroids and infertility. Their results support the hypothesis that fibroid-associated infertility could be potentially caused by alterations in the BMP-pathway in the endometrium, possibly leading to disturbed angiogenesis and reducing the success of implantation. This is in line with published reviews about implantation failure [77,78,79]. Our review shows increased levels of eNOS in the endometrium of patients with fibroids and infertility. As eNOS is important in continuing pregnancy and uterine quiescence during implantation, this supports the hypotheses that fibroids can impair implantation or increase the risk of miscarriage [49]. Remarkably, in patients with fibroids and AUB, eNOS was also significantly increased [35,80], supporting the idea that eNOS expression is increased in the endometrium due to the presence of fibroids. Unfortunately, there were no studies evaluating vascular morphology in the endometrium of women with fibroids in relation to fertility.

### 4.2. Strengths and Limitations

This systematic review discusses angiogenesis in the endometrium of patients with fibroids and evaluates whether altered angiogenesis may influence the symptoms of AUB or infertility. It is the first systematic review that addresses the effects of several pharmaceutical therapies, such as continuous OCP, GnRHa, and UPA therapy on (disrupted) angiogenesis in the endometrium of patients with fibroids. The strengths of this review comprise the registration in the PROSPERO International prospective register of systematic reviews, the systematic approach following PRISMA guidelines for systematic reviews, the conducted search in four databases, and the use of internationally accepted checklists to assess the RoB for included articles. Another strength of this review is the structural way it presents the broad range of possibly involved angiogenic parameters and morphological vessel characteristics. Nonetheless, the review also has some limitations. RoB analyses showed that only five of the 15 included studies were of good quality. Another limitation was the lack of objective scoring of AUB, as most included studies did not even describe how AUB was objectified. In total, three studies did not describe any fibroid complaints. Therefore, they were presented in this review as a separate group of fibroid patients without AUB, but we cannot exclude that these patients did not suffer from any complaints as their endometrium samples were extracted from a database. Different comparisons with control groups (AUB or NMB) were also made, which makes it difficult to compare the presented outcomes of these studies. In addition, the compared groups showed great heterogeneity in terms of studied markers, moment of collection of the endometrial sample in the menstrual cycle, and treatment types and exposure time. Pharmaceutical therapy also comprised different medicines, including continuous OCP for a broad range of exposure (1–24 months) and GnRHa and UPA for short time exposure (3–6 months). Moreover, most of the parameters were only studied in one or two publications in small patient groups.

## 5. Conclusions

The increased expression of pro-angiogenic factors in the endometrium of patients with fibroids and/or AUB-L support our hypothesis that angiogenesis is impaired in these patients, leading to more fragile, dilated, and leaking blood vessels. Furthermore, this review suggests that pharmaceutical therapy for fibroids influences this altered expression. Pharmaceutical therapy can therefore decrease AUB complaints, as it appears to reverse the initiated alteration of angiogenic factors. Moreover, the identified changes in angiogenic factors could indicate altered angiogenesis in the endometrium of patients with fibroids, and these may play an intermediate role between the presence of fibroids and infertility. However, as the observed limitations indicate, more research is needed to draw hard conclusions.

## Figures and Tables

**Table 1:** Characteristics of the different types of angiogenesis [18].

	A. Sprouting Angiogenesis	B. Intussusceptive Angiogenesis	C. Vessel Elongation	D. Coalescent Angiogenesis
Process	Vessel growth from existing vessels by initiation of tip cells	An initial vessel splits into two daughter vessels	Passive cell growth, whereas a vessel widens and/or extends	Capillary mesh fusion by parallel processes of vessel maturation and regression
Stimulant	Hypoxia, growth factors	Growth factors, shear stress	Metabolic signals of surrounding cells	Shear stress
Vessel perfusion	Only after completion	Continuous	Continuous	Continuous
Growth rate	Slow	Fast	Fast	Fast
(Possible) Effect on vascular morphology ^a^	↑ MVD	↑ MVD	=/↑ MVD	=/↓ MVD ↑ Vessel diameter ^b^

^a^↑/ ↓/= indicating respectively an increase, decrease or no change, in microvessel density (MVD) or vessel diameter due to this angiogenic mechanism = indicating no difference in in microvessel density (MVD) with this angiogenic mechanism. ^b^ The outcome that the MVD is unchanged or decreased in coalescent angiogenesis is reasoned by the authors, based on the scarce evidence for this form of angiogenesis.

**Table 2:** Inclusion and exclusion criteria of Search I and II.

Search I	Inclusion Criteria	Exclusion Criteria
Population	Premenopausal patients With fibroids and/or AUB With and without of exogenous hormones	Uterine abnormalities other than fibroid(s).
Intervention		In vitro experiment with non-human cells
Outcomes	Angiogenic factors in endometrium Vascular characteristics	Menstrual effluent Serum levels
Study design	Randomized Controlled Trial Case control Cohort study	Meta-analysis Case reports Reviews
**Search II**	**Inclusion Criteria**	**Exclusion Criteria**
Population	Premenopausal patients With fibroids With infertility	Uterine abnormalities other than fibroid(s).
Intervention		In vitro experiment with non-human cells
Outcomes	Angiogenic factors in endometrium Vascular characteristics	Menstrual effluent Serum levels
Study design	Randomized controlled trial Case control Cohort study	Meta-analysis Case reports Reviews

**Table 4:** Outcomes of the included articles regarding angiogenesis in the endometrium of patients with uterine fibroids (UF) compared to normal controls and UF with abnormal uterine blood loss (AUB) compared with normal controls.

	Uterine Fibroids (UF) Compared to Normal Controls	Abnormal Uterine Blood (AUB) Loss and UF Compared to Normal Controls		Studies [Reference Citation Number]
***Angiogenic parameters***	*Outcomes ^a^*	*Outcomes ^a^ in the* *proliferative phase*	*Outcomes ^a^ in the* *secretory phase*	
ADM	↑			Hague et al. 2000 [38]
aFGF	=			Hague et al. 2000 [38]
bFGF	=			Hague et al. 2000 [38]
COX1	= ^b^			Governini et al. 2021 [46]
COX2	= ^b^			Governini et al. 2021 [46]
eNOS		↑	↑ ^c^	Oh et al. 2013 [35]
FGF-R1		=	↑^a^	Anania et al. 1997 [39]
IL-1β/6/10	↓			Governini et al. 2021 [46]
STAT3	= ^b^			Governini et al. 2021 [46]
VEGF	↑/= ^b^	↑	↑ ^d^	**UF**: Hague et al. 2000 [38] Governini et al. 2021 [46] **AUB & UF**: Zhang et al. 2010 [40]
***Vascular morphology outcomes ^a^***				
MVD (by H&E) ^e^	=	=		**UF**: Hague et al. 2000 [38] **AUB & UF**: Makhija et al. 2008 [34]
Vessel diameter		=		Makhija et al. 2008 [34]
Venous lakes	↑			Bereza et al. 2014 [45]
Vascular congestion		=		Makhija et al. 2008 [34]

^a^ ↑ indicating an increased expression of angiogenic parameters or vascular morphology outcomes in patients with uterine fibroids compared to normal controls; ↓ indicating a decreased expression and/or outcome in patients with uterine fibroids compared to normal controls; = indicating no difference in expression and/or outcome in patients with uterine fibroids compared to normal controls. ^b^ Angiogenic factors were increased, however, not significantly increased. ^c^ Significantly higher expression in patients with UF and even significantly higher expression of eNOS in symptomatic fibroids. ^d^ Increased VEGF expression was seen in patients with UF in comparison with normal controls, with even higher VEGF expression in symptomatic UF. ^e^ H&E: hematoxylin and osin.

**Table 5:** Outcomes of the included studies that compared the effect of pharmaceutical therapy (study group) on angiogenesis in the endometrium of patients with uterine fibroids with AUB or without AUB (control groups).

Angiogenic Parameters ^a^			Studies ^b^ [Reference Citation Number]
**Comparison: patients with AUB and UF with and without continuous Gestinol ^b,c^**			
*Angiogenic factor*		*Outcomes ^d^*	
VEGF	↓		Maia et al. 2008 [36]
Aromatase	↓ ^f^		Maia et al. 2008 [36]
COX-2 A vs. C ^b^ BTB vs. C ^b^ BTB vs. no MBL ^b,e^	*Proliferative phase*	*Secretory phase*	Maia et al. 2008 [36] Maia et al. 2008 [36] Maia et al. 2008 [36]
	↓	=	
	=	↑	
	↑		
**Comparison: patients with AUB, UF and GnRHa compared to patients with AUB and without UF and GnRHa ^b,c^**			
*Vessel*		*Outcomes ^d^*	
MVD (vWF)	↓		Khan et al. 2010a [42]
Macrophages infiltration (CD68)	↓		Khan et al. 2010a [42]
MCP-1	↓		Khan et al. 2010a [42]
*Proliferation (Ki67)*			
Endothelial cell	↓		Khan et al. 2010b [41]
Epithelial and stromal cell	↓		Khan et al. 2010b [41]
**Comparison: patients with AUB, UF and UPA compared to patients with NMB and without UF and UPA ^b,c^**			
*Angiogenic factor*		*Outcomes ^d^*	
VEGFR2	=		Kolanska et al. 2019 [37]
ER	↑		Kolanska et al. 2019 [37]
PR	↑		Kolanska et al. 2019 [37]
*Proliferation (Ki67)*	*Proliferative phase*	*Secretory phase*	
Epithelial cell	↓ ^g^	↑ ^g^	Kolanska et al. 2019 [37]
Stromal cell	↓ ^g^	= ^g^	Kolanska et al. 2019 [37]

^a^ ER: estradiol receptor; MVD: micro vessel density; PR: progesterone receptor; VEGF: vascular endothelial growth (-R: receptor); vWF: von Willebrand factor; ^b^ A: amenorrhea; AUB: abnormal uterine bleeding; BTB: break-through-bleeding; HMB: heavy menstrual bleeding; NMB: normal menstrual bleeding; UF: uterine fibroids; ^c^ Gestinol: 75 mcg gestodene + 30 mcg ethinylestradiol; GnRHa: gonadotropin-releasing hormone agonist; UPA: Ulipristal acetate; ^d^ ↑ indicating an increased expression of angiogenic parameters or vascular morphology outcomes in patients with uterine fibroids compared to normal controls; ↓ indicating a decreased expression and/or outcome in patients with uterine fibroids compared to normal controls; = indicating no difference in expression and/or outcome in patients with uterine fibroids compared to normal controls; ^e^ Compared between both Gestinol users; ^f^ Significant in proliferative and early luteal phase of the cycle, not significant in late luteal phase; ^g^ Results for the superficial layer of the endometrium, UPA showed no effect on proliferation in the basal layer of the endometrium.

**Table 6:** Outcomes of the included articles regarding angiogenesis in patients with fibroids and infertility.

Angiogenic Arameters ^a^	Outcomes ^b^	Studies [Reference Citation Number]
BMP-2	↓	Sinclair et al. 2011 [50]
BMPR-1A	= ↓	Doherty et al. 2015 [47] Sinclair et al. 2011 [50]
BMPR-1B	↓	Doherty et al. 2015, Sinclair et al. 2011 [47,50]
BMPR-2	↓	Doherty et al. 2015, Sinclair et al. 2011 [47,50]
CLDN-5	↓	Kozachenko et al. 2020 [48]
eNOS	↑	Novin et al. 2018 [49]
Estradiol receptor (ER)	↓	Kozachenko et al. 2020 [48]
HOXA10	↓ =	Doherty et al. 2015 [47] Kozachenko et al. 2020 [48]
HOXA11	↓	Kozachenko et al. 2020 [48]
Integrin αVβ3	↓	Kozachenko et al. 2020 [48]
LIF	↓	Doherty et al. 2015, Kozachenko et al. 2020 [47,48]
Progesterone receptor (PR)	↓	Kozachenko et al. 2020 [48]
SPEI (PR/ER)	=	Kozachenko et al. 2020 [48]
VEGF-A	↓	Kozachenko et al. 2020 [48]

^a^ BMP: bone morphogenetic protein (-R: receptor); CLDN-5: Claudin-5; eNOS: endothelial isoform of nitric oxide synthase; HOXA: Homeobox-A; LIF: leukemia inhibitory factor; SPEI: stromal progesterone-estrogen index; VEGF-A: vascular endothelial growth factor-A. ^b^ ↑ indicating an increased expression of angiogenic parameters or vascular morphology outcomes in patients with uterine fibroids compared to normal controls; ↓ indicating a decreased expression and/or outcome in patients with uterine fibroids compared to normal controls; = indicating no difference in expression and/or outcome in patients with uterine fibroids compared to normal controls.

## Data Availability

Not applicable.

## Supplementary Materials

The following supporting information can be downloaded at: https://www.mdpi.com/article/10.3390/ijms24087011/s1.

## References

[1] Selo-Ojeme D., Lawal O., Shah J., Mandal R., Pathak S., Selo-Ojeme U., Samuel D. (2008). The incidence of uterine leiomyoma and other pelvic ultrasonographic findings in 2,034 consecutive women in a north London hospital. J. Obstet. Gynaecol..

[2] Stewart E.A., Laughlin-Tommaso S.K., Catherino W.H., Lalitkumar S., Gupta D., Vollenhoven B. (2016). Uterine fibroids. Nat. Rev. Dis. Primers.

[3] Parker W.H. (2007). Etiology, symptomatology, and diagnosis of uterine myomas. Fertil. Steril..

[4] Farquhar C.M., Steiner C.A. (2002). Hysterectomy rates in the United States 1990–1997. Obstet. Gynecol..

[5] Wright K.N., Jonsdottir G.M., Jorgensen S., Shah N., Einarsson J.I. (2012). Costs and outcomes of abdominal, vaginal, laparoscopic and robotic hysterectomies. JSLS.

[6] Munro M.G., Critchley H.O., Broder M.S., Fraser I.S., Disorders F.W.G.o.M. (2011). FIGO classification system (PALM-COEIN) for causes of abnormal uterine bleeding in nongravid women of reproductive age. Int. J. Gynaecol. Obstet..

[7] Munro M.G., Critchley H.O.D., Fraser I.S., Committee F.M.D. (2018). The two FIGO systems for normal and abnormal uterine bleeding symptoms and classification of causes of abnormal uterine bleeding in the reproductive years: 2018 revisions. Int. J. Gynaecol. Obstet..

[8] National Collaborating Centre for Centre for Women’s and Children’s Health (2007). National Institute for Health and Clinical Excellence: Guidance. Heavy Menstrual Bleeding.

[9] NICE (2018). NICE Guideline: Heavy Menstrual Bleeding: Assessment and Management.

[10] Navarro A., Bariani M.V., Yang Q., Al-Hendy A. (2021). Understanding the Impact of Uterine Fibroids on Human Endometrium Function. Front. Cell. Dev. Biol..

[11] Cetin E., Al-Hendy A., Ciebiera M. (2020). Non-hormonal mediators of uterine fibroid growth. Curr. Opin. Obstet. Gynecol..

[12] Deligdish L., Loewenthal M. (1970). Endometrial changes associated with myomata of the uterus. J. Clin. Pathol..

[13] Buttram V.C., Reiter R.C. (1981). Uterine leiomyomata: Etiology, symptomatology, and management. Fertil. Steril..

[14] Dudley A.C., Griffioen A.W. (2023). Targeting pathological angiogenesis: Mechanisms and therapeutic strategies. Angiogenesis.

[15] Yetkin-Arik B., Kastelein A.W., Klaassen I., Jansen C., Latul Y.P., Vittori M., Biri A., Kahraman K., Griffioen A.W., Amant F. (2021). Angiogenesis in gynecological cancers and the options for anti-angiogenesis therapy. Biochim. Biophys. Acta Rev. Cancer.

[16] Latacz E., Caspani E., Barnhill R., Lugassy C., Verhoef C., Grunhagen D., Van Laere S., Fernandez Moro C., Gerling M., Dirix M. (2020). Pathological features of vessel co-option versus sprouting angiogenesis. Angiogenesis.

[17] Rogers P.A., Gargett C.E. (1998). Endometrial angiogenesis. Angiogenesis.

[18] Nitzsche B., Rong W.W., Goede A., Hoffmann B., Scarpa F., Kuebler W.M., Secomb T.W., Pries A.R. (2022). Coalescent angiogenesis-evidence for a novel concept of vascular network maturation. Angiogenesis.

[19] Adair T.H., Montani J.P. (2010). Overview of Angiogenesis. Angiogenesis.

[20] Gambino L.S., Wreford N.G., Bertram J.F., Dockery P., Lederman F., Rogers P.A. (2002). Angiogenesis occurs by vessel elongation in proliferative phase human endometrium. Hum. Reprod..

[21] Pezzella F., Kerbel R.S. (2022). On coalescent angiogenesis and the remarkable flexibility of blood vessels. Angiogenesis.

[22] Girling J.E., Rogers P.A. (2005). Recent advances in endometrial angiogenesis research. Angiogenesis.

[23] Möller B., Rasmussen C.H., Lindblom B., Olovsson M. (2001). Expression of the angiogenic growth factors VEGF, FGF-2, EGF and their receptors in normal human endometrium during the menstrual cycle. Mol. Hum. Reprod..

[24] Burton G.J., Charnock-Jones D.S., Jauniaux E. (2009). Regulation of vascular growth and function in the human placenta. Reproduction.

[25] Middelkoop M.A., Don E.E., Hehenkamp W.J.K., Polman N.J., Griffioen A.W., Huirne J.A.F. (2023). Angiogenesis in abnormal uterine bleeding: A narrative review. Hum. Reprod. Update.

[26] Harmsen M.J., Wong C.F.C., Mijatovic V., Griffioen A.W., Groenman F., Hehenkamp W.J.K., Huirne J.A.F. (2019). Role of angiogenesis in adenomyosis-associated abnormal uterine bleeding and subfertility: A systematic review. Hum. Reprod. Update.

[27] Liberati A., Altman D.G., Tetzlaff J., Mulrow C., Gøtzsche P.C., Ioannidis J.P.A., Clarke M., Devereaux P.J., Kleijnen J., Moher D. (2009). The PRISMA statement for reporting systematic reviews and meta-analyses of studies that evaluate healthcare interventions: Explanation and elaboration. BMJ.

[28] Jørgensen L., Paludan-Müller A.S., Laursen D.R., Savović J., Boutron I., Sterne J.A., Higgins J.P., Hróbjartsson A. (2016). Evaluation of the Cochrane tool for assessing risk of bias in randomized clinical trials: Overview of published comments and analysis of user practice in Cochrane and non-Cochrane reviews. Syst. Rev..

[29] Stang A. (2010). Critical evaluation of the Newcastle-Ottawa scale for the assessment of the quality of nonrandomized studies in meta-analyses. Eur. J. Epidemiol..

[30] Sterne J.A.C., Savovic J., Page M.J., Elbers R.G., Blencowe N.S., Boutron I., Cates C.J., Cheng H.Y., Corbett M.S., Eldridge S.M. (2019). RoB 2: A revised tool for assessing risk of bias in randomised trials. BMJ.

[31] Wells G.A., Shea B., O’Conell D., Peterson J., Welch V., Losos M., Tugwell P. The Newcastle-Ottawa Scale (NOS) for assessing the quality of nonrandomised studies in meta-analyses. http://www.ohri.ca/programs/clinical_epidemiology/oxford.asp.

[32] Press W., World Health Organization (2006). Reproductive Health Indicators—Guidelines for Their Generation, Interpretation and Analysis for Global Monitoring.

[33] Moher D., Liberati A., Tetzlaff J., Altman D.G., Group P. (2009). Preferred reporting items for systematic reviews and meta-analyses: The PRISMA statement. PLoS Med..

[34] Makhija D., Mathai A.M., Naik R., Kumar S., Rai S., Pai M.R., Baliga P. (2008). Morphometric evaluation of endometrial blood vessels. Indian. J. Pathol. Microbiol..

[35] Oh N.J., Ryu K.Y., Jung C.N., Yi S.Y., Kim S.R. (2013). Expression of endothelial nitric oxide synthase in the uterus of patients with leiomyoma or adenomyosis. J. Obstet. Gynaecol. Res..

[36] Maia H., Casoy J., Pimentel K., Correia T., Athayde C., Cruz T., Coutinho E.M. (2008). Effect of oral contraceptives on vascular endothelial growth factor, Cox-2 and aromatase expression in the endometrium of uteri affected by myomas and associated pathologies. Contraception.

[37] Kolanska K., Varinot J., Canlorbe G., Bergeron C., Mekinian A., Capmas P., Koskas M., Darai E., Aractingi S., Bendifallah S. (2019). Absence of predictable long-term molecular effect of ulipristal acetate (UPA) on the endometrium. Reprod. Biomed. Online.

[38] Hague S., Zhang L., Oehler M.K., Manek S., MacKenzie I.Z., Bicknell R., Rees M.C. (2000). Expression of the hypoxically regulated angiogenic factor adrenomedullin correlates with uterine leiomyoma vascular density. Clin. Cancer Res..

[39] Anania C.A., Stewart E.A., Quade B.J., Hill J.A., Nowak R.A. (1997). Expression of the fibroblast growth factor receptor in women with leiomyomas and abnormal uterine bleeding. Mol. Hum. Reprod..

[40] Zhang Y.B., Zhu S.J., Jia W.J. (2010). Expressions and significance of VEGF and MMP-2 in endometrium of patients with sympotomatic uterine leiomyomas. Chin. Journal. Cancer Prev. Treat..

[41] Khan K.N., Kitajima M., Hiraki K., Fujishita A., Nakashima M., Ishimaru T., Masuzaki H. (2010). Cell proliferation effect of GnRH agonist on pathological lesions of women with endometriosis, adenomyosis and uterine myoma. Hum. Reprod..

[42] Khan K.N., Kitajima M., Hiraki K., Fujishita A., Sekine I., Ishimaru T., Masuzaki H. (2010). Changes in tissue inflammation, angiogenesis and apoptosis in endometriosis, adenomyosis and uterine myoma after GnRH agonist therapy. Hum. Reprod..

[43] Noyes R.W., Hertig A.T., Rock J. (2019). Reprint of: Dating the Endometrial Biopsy. Fertil. Steril..

[44] Fox H., Buckley C.H., Gresham G.A. (1983). Endometrium. Atlas of Gynaecological Pathology.

[45] Bereza T., Tomaszewski K.A., Lis G.J., Mizia E., Pasternak A., Mazur M., Mituś J. (2014). ‘Venous lakes’—A corrosion cast scanning electron microscopy study of regular and myomatous human uterine blood vessels. Folia Morphol..

[46] Governini L., Marrocco C., Semplici B., Pavone V., Belmonte G., Luisi S., Petraglia F., Luddi A., Piomboni P. (2021). Extracellular matrix remodeling and inflammatory pathway in human endometrium: Insights from uterine leiomyomas. Fertil. Steril..

[47] Doherty L.F., Taylor H.S. (2015). Leiomyoma-derived transforming growth factor-beta impairs bone morphogenetic protein-2-mediated endometrial receptivity. Fertil. Steril..

[48] Kozachenko I.F., Faizullina N.M., Shchegolev A.I., Adamyan L.V. (2020). Endometrial receptivity in patients with benign uterine diseases and infertility before and after surgery. Akusherstvo Ginekol./Obstet. Gynecol..

[49] Novin M.G., Moini A., Niknezhad S., Najafi T., Novin M.G. (2018). Immunohistochemical localization of endothelial nitric oxide synthase in endometrial tissues of women with uterine myomas. Int. J. Women’s Health Reprod. Sci..

[50] Sinclair D.C., Mastroyannis A., Taylor H.S. (2011). Leiomyoma simultaneously impair endometrial BMP-2-mediated decidualization and anticoagulant expression through secretion of TGF-beta3. J. Clin. Endocrinol. Metab..

[51] Hillen F., Griffioen A.W. (2007). Tumour vascularization: Sprouting angiogenesis and beyond. Cancer Metastasis Rev..

[52] Khorram O., Garthwaite M., Magness R.R. (1999). Endometrial and myometrial expression of nitric oxide synthase isoforms in pre- and postmenopausal women. J. Clin. Endocrinol. Metab..

[53] Wong H.K., Cheung T.T., Cheung B.M. (2012). Adrenomedullin and cardiovascular diseases. JRSM Cardiovasc. Dis..

[54] Griffioen A.W., Molema G. (2000). Angiogenesis: Potentials for pharmacologic intervention in the treatment of cancer, cardiovascular diseases, and chronic inflammation. Pharmacol. Rev..

[55] Lai T.H., Vlahos N., Shih Ie M., Zhao Y. (2015). Expression Patterns of VEGF and Flk-1 in Human Endometrium during the Menstrual Cycle. J. Reprod. Infertil..

[56] Kitamura K., Kangawa K., Kawamoto M., Ichiki Y., Nakamura S., Matsuo H., Eto T. (1993). Adrenomedullin: A novel hypotensive peptide isolated from human pheochromocytoma. Biochem. Biophys. Res. Commun..

[57] Nikitenko L.L., MacKenzie I.Z., Rees M.C., Bicknell R. (2000). Adrenomedullin is an autocrine regulator of endothelial growth in human endometrium. Mol. Hum. Reprod..

[58] Nikitenko L.L., Smith D.M., Hague S., Wilson C.R., Bicknell R., Rees M.C. (2002). Adrenomedullin and the microvasculature. Trends Pharmacol. Sci..

[59] Ha C., Stavreus-Evers A., Landgren B.M., Mints M., Rees M.C. (2009). Adrenomedullin and its receptor, calcitonin receptor-like receptor, are aberrantly expressed in women with idiopathic menorrhagia. Mol. Med. Rep..

[60] Smith O.P., Jabbour H.N., Critchley H.O. (2007). Cyclooxygenase enzyme expression and E series prostaglandin receptor signalling are enhanced in heavy menstruation. Hum. Reprod..

[61] Richards E.G., El-Nashar S.A., Schoolmeester J.K., Keeney G.L., Mariani A., Hopkins M.R., Dowdy S.C., Daftary G.S., Famuyide A.O. (2017). Abnormal Uterine Bleeding Is Associated With Increased BMP7 Expression in Human Endometrium. Reprod. Sci..

[62] Tian M., Neil J.R., Schiemann W.P. (2011). Transforming growth factor-beta and the hallmarks of cancer. Cell. Signal..

[63] Luddi A., Marrocco C., Governini L., Semplici B., Pavone V., Capaldo A., Tosti C., Greco S., Luisi S., Ciarmela P. (2019). Increased expression of neurogenic factors in uterine fibroids. Hum. Reprod..

[64] Buxton I.L. (2004). Regulation of uterine function: A biochemical conundrum in the regulation of smooth muscle relaxation. Mol. Pharmacol..

[65] Ota H., Igarashi S., Hatazawa J., Tanaka T. (1998). Endothelial nitric oxide synthase in the endometrium during the menstrual cycle in patients with endometriosis and adenomyosis. Fertil. Steril..

[66] Najafi T., Novin M.G., Ghazi R., Khorram O. (2012). Altered endometrial expression of endothelial nitric oxide synthase in women with unexplained recurrent miscarriage and infertility. Reprod. Biomed. Online.

[67] Avraamides C.J., Garmy-Susini B., Varner J.A. (2008). Integrins in angiogenesis and lymphangiogenesis. Nat. Rev. Cancer.

[68] Hodivala-Dilke K. (2008). αvβ3 integrin and angiogenesis: A moody integrin in a changing environment. Curr. Opin. Cell. Biol..

[69] Lee K.Y., Jeong J.W., Wang J., Ma L., Martin J.F., Tsai S.Y., Lydon J.P., DeMayo F.J. (2007). Bmp2 is critical for the murine uterine decidual response. Mol. Cell. Biol..

[70] Smith S.K. (2001). Regulation of angiogenesis in the endometrium. Trends Endocrinol. Metab..

[71] Godoy Morales H.S.G.H., Paz Martinez A.d.J.P.A., Mamani Cancino A.D.M.A., Lozano Sánchez J.M.L.J., Romo Aguirre C.R.C., Montaño L.F.M.L. (2013). Claudin 5 as a marker of endometrial receptivity in patients with previous implantation failure. Fertil. Steril..

[72] Hlatky L., Hahnfeldt P., Folkman J. (2002). Clinical Application of Antiangiogenic Therapy: Microvessel Density, What It Does and Doesn’t Tell Us. JNCI J. Natl. Cancer Inst..

[73] Jabbour H.N., Kelly R.W., Fraser H.M., Critchley H.O. (2006). Endocrine regulation of menstruation. Endocr. Rev..

[74] Donoghue J.F., McGavigan C.J., Lederman F.L., Cann L.M., Fu L., Dimitriadis E., Girling J.E., Rogers P.A. (2012). Dilated thin-walled blood and lymphatic vessels in human endometrium: A potential role for VEGF-D in progestin-induced break-through bleeding. PLoS ONE.

[75] Ravet S., Munaut C., Blacher S., Brichant G., Labied S., Beliard A., Chabbert-Buffet N., Bouchard P., Foidart J.M., Pintiaux A. (2008). Persistence of an intact endometrial matrix and vessels structure in women exposed to VA-2914, a selective progesterone receptor modulator. J. Clin. Endocrinol. Metab..

[76] Frijlingh M., De Milliano I., Hehenkamp W.J.K., Huirne J.A.F. (2020). Differences in fibroid vascularity after three months of pre-treatment with leuprolide acetate or ulipristal acetate: A pilot study. Eur. J. Obstet. Gynecol. Reprod. Biol..

[77] Horne A.W., Critchley H.O. (2007). The effect of uterine fibroids on embryo implantation. Semin. Reprod. Med..

[78] Cakmak H., Taylor H.S. (2011). Implantation failure: Molecular mechanisms and clinical treatment. Hum. Reprod. Update.

[79] Donaghay M., Lessey B.A. (2007). Uterine receptivity: Alterations associated with benign gynecological disease. Semin. Reprod. Med..

[80] Blumenthal R.D., Taylor A.P., Goldman L., Brown G., Goldenberg D.M. (2002). Abnormal expression of the angiopoietins and Tie receptors in menorrhagic endometrium. Fertil. Steril..

