# Supplementary material for: Endometrial Angiogenesis of Abnormal Uterine Bleeding and Infertility in Patients with Uterine Fibroids—A Systematic Review
Source: Int J Mol Sci. 2023 Apr 10;24(8):7011. doi: 10.3390/ijms24087011 (PMC10138959; doi:10.3390/ijms24087011)
Supplement: Supplementary file 1 [file ijms-24-07011-s001.zip › ijms-2287867-supplementary.pdf]

Supplementary file S1 – Search strategy

Search 1: Role of angiogenesis in endometrium of patients with uterine fibroids and AUB

1. Fibroids
2. Angiogenesis
3. Abnormal uterine bleeding

PubMed Session Results (16 Jun 2022)

#3	**#1 AND #2**	979
#2	"Myoma"[Mesh:NoExp] OR "Leiomyoma"[Mesh] OR "myoma*"[tiab] OR "leiomyoma*"[tiab] OR "fibroid*"[tiab] OR "fibroma*"[tiab] OR "fibromyoma*"[tiab] OR "angiomyoma*"[tiab] OR "angioleiomyoma*"[tiab]	45,966
#1	"Endothelium, Vascular"[Mesh] OR "Microvessels"[Mesh:NoExp] OR "Capillaries"[Mesh] OR "Pericytes"[Mesh] OR "Angiogenic Proteins"[Mesh] OR "Angiogenesis Modulating Agents"[Mesh] OR "Neovascularization, Physiologic"[Mesh] OR "Neovascularization, Pathologic"[Mesh] OR "Receptors, Fibroblast Growth Factor"[Mesh:NoExp] OR "Receptor, Fibroblast Growth Factor, Type 1"[Mesh] OR "Receptor, Fibroblast Growth Factor, Type 2"[Mesh] OR "Receptor, Fibroblast Growth Factor, Type 4"[Mesh] OR "Fibroblast Growth Factors"[Mesh] OR "Platelet-Derived Growth Factor"[Mesh] OR "Transforming Growth Factor alpha"[Mesh] OR "Transforming Growth Factor beta"[Mesh] OR "Adrenomedullin"[Mesh] OR "pericyt*"[tiab] OR "vascular endothel*"[tiab] OR "capillary endothel*"[tiab] OR "angioprotein*"[tiab] OR "angiogenesis"[tiab] OR "antiangiogenesis"[tiab] OR "neoangiogenesis"[tiab] OR "angiogenetic"[tiab] OR "antiangiogenetic"[tiab] OR "angiogenic"[tiab] OR "antiangiogenic"[tiab] OR "angiogenin"[tiab] OR "angiostatic"[tiab] OR "neovasculari*"[tiab] OR "neo-vasculari*"[tiab] OR "angiopoietin*"[tiab] OR "angio-poietin*"[tiab] OR "angiostatin*"[tiab] OR "angio-statin*"[tiab] OR "endostatin*"[tiab] OR "vegf*"[tiab] OR "Vascular Endothelial Growth Factor*"[tiab] OR "Vasculotropin"[tiab] OR "c-fos-Induced Growth Factor*"[tiab] OR "figf"[tiab] OR "hif-1*"[tiab] OR "hif1*"[tiab] OR "microvessel density"[tiab] OR "micro vessel density"[tiab] OR "MVD"[tiab] OR "bloodvessel density"[tiab] OR "blood vessel density"[tiab] OR "BVD"[tiab] OR "vessel fragility"[tiab] OR "vascular fragility"[tiab] OR "vessel wall*"[tiab] OR "VSMC"[tiab] OR "vascular smooth muscle cell*"[tiab] OR "HB-EGF"[tiab] OR "FGF"[tiab] OR "FGFR*"[tiab] OR "fibroblast growth factor"[tiab] OR "PDGF"[tiab] OR "platelet derived growth factor"[tiab] OR "Transforming Growth Factor*"[tiab] OR "TGF"[tiab] OR "adrenomedullin"[tiab] OR "arteriogenes*"[tiab] OR "vasculogenes*"[tiab] OR "vasculo-genes*"[tiab]	609,829

Embase.com Session Results (16 Jun 2022)

#3	**#1 AND #2**	2,354

#2	'myoma'/de OR 'leiomyoma'/exp OR 'uterus myoma'/exp OR myoma*:ab,ti,kw OR leiomyoma*:ab,ti,kw OR fibroid*:ab,ti,kw OR fibroma*:ab,ti,kw OR fibromyoma*:ab,ti,kw OR angiomyoma*:ab,ti,kw OR angioleiomyoma*:ab,ti,kw	66,869
#1	'vascular endothelium'/exp OR 'microvasculature'/de OR 'capillary'/de OR 'capillary endothelium'/de OR 'pericyte'/de OR 'angiogenesis'/exp OR 'neovascularization (pathology)'/de OR 'fibroblast growth factor receptor'/exp OR 'fibroblast growth factor receptor 1'/exp OR 'fibroblast growth factor receptor 2'/exp OR 'fibroblast growth factor receptor 4'/exp OR 'adrenomedullin'/exp OR 'angiogenesis modulator'/exp OR 'angiogenic protein'/exp OR 'angiogenin'/exp OR 'angiopoietin'/exp OR 'angiopoietin 1'/exp OR 'angiopoietin 2'/exp OR 'endothelial cell growth factor'/exp OR 'fibroblast growth factor'/exp OR 'fibroblast growth factor 1'/exp OR 'fibroblast growth factor 10'/exp OR 'fibroblast growth factor 2'/exp OR 'platelet derived growth factor'/exp OR 'platelet derived growth factor A'/exp OR 'platelet derived growth factor AA'/exp OR 'platelet derived growth factor AB'/exp OR 'platelet derived growth factor B'/exp OR 'platelet derived growth factor BB'/exp OR 'platelet derived growth factor C'/exp OR 'platelet derived growth factor D'/exp OR 'transforming growth factor alpha'/exp OR 'transforming growth factor beta'/exp OR 'vasculotropin'/exp OR 'vasculotropin 121'/exp OR 'vasculotropin 165'/exp OR 'vasculotropin A'/exp OR 'vasculotropin B'/exp OR pericyt*:ab,ti,kw OR 'vascular endothel*':ab,ti,kw OR 'capillary endothel*':ab,ti,kw OR angioprotein*:ab,ti,kw OR angiogenesis:ab,ti,kw OR antiangiogenesis:ab,ti,kw OR neoangiogenesis:ab,ti,kw OR angiogenetic:ab,ti,kw OR antiangiogenetic:ab,ti,kw OR angiogenic:ab,ti,kw OR antiangiogenic:ab,ti,kw OR angiogenin:ab,ti,kw OR angiostatic:ab,ti,kw OR neovasculari*:ab,ti,kw OR 'neo-vasculari*':ab,ti,kw OR angiopoietin*:ab,ti,kw OR 'angio-poietin*':ab,ti,kw OR angiostatin*:ab,ti,kw OR 'angio-statin*':ab,ti,kw OR endostatin*:ab,ti,kw OR vegf*:ab,ti,kw OR 'Vascular Endothelial Growth Factor*':ab,ti,kw OR Vasculotropin:ab,ti,kw OR 'c-fos-Induced Growth Factor*':ab,ti,kw OR figf:ab,ti,kw OR 'hif-1*':ab,ti,kw OR hif1*:ab,ti,kw OR 'microvessel density':ab,ti,kw OR 'micro vessel density':ab,ti,kw OR MVD:ab,ti,kw OR 'bloodvessel density':ab,ti,kw OR 'blood vessel density':ab,ti,kw OR BVD:ab,ti,kw OR 'vessel fragility':ab,ti,kw OR 'vascular fragility':ab,ti,kw OR 'vessel wall*':ab,ti,kw OR VSMC:ab,ti,kw OR 'vascular smooth muscle cell*':ab,ti,kw OR 'HB-EGF':ab,ti,kw OR FGF:ab,ti,kw OR FGFR*:ab,ti,kw OR 'fibroblast growth factor':ab,ti,kw OR PDGF:ab,ti,kw OR 'platelet derived growth factor':ab,ti,kw OR 'Transforming Growth Factor*':ab,ti,kw OR TGF:ab,ti,kw OR adrenomedullin:ab,ti,kw OR arteriogenes*:ab,ti,kw OR vasculogenes*:ab,ti,kw OR 'vasculo-genes*':ab,ti,kw	1,183,130

Web of Science Session Results (16 Jun 2022)

#3	**#1 AND #2**	1,107
#2	TS=("myoma*" OR "leiomyoma*" OR "fibroid*" OR "fibroma*" OR "fibromyoma*" OR "angiomyoma*" OR "angioleiomyoma*")	38,134
#1	TS=("pericyt*" OR "vascular endothel*" OR "capillary endothel*" OR "angioprotein*" OR "angiogenesis" OR "antiangiogenesis" OR "neoangiogenesis" OR "angiogenetic" OR "antiangiogenetic" OR "angiogenic" OR "antiangiogenic" OR "angiogenin" OR "angiostatic" OR	608,677

	"neovasculari*" OR "neo-vasculari*" OR "angiopoietin*" OR "angio-poietin*" OR "angiostatin*" OR "angio-statin*" OR "endostatin*" OR "vegf*" OR "Vascular Endothelial Growth Factor*" OR "Vasculotropin" OR "c-fos-Induced Growth Factor*" OR "figf" OR "hif-1*" OR "hif1*" OR "microvessel density" OR "micro vessel density" OR "MVD" OR "bloodvessel density" OR "blood vessel density" OR "BVD" OR "vessel fragility" OR "vascular fragility" OR "vessel wall*" OR "VSMC" OR "vascular smooth muscle cell*" OR "HB-EGF" OR "FGF" OR "FGFR*" OR "fibroblast growth factor" OR "PDGF" OR "platelet derived growth factor" OR "Transforming Growth Factor*" OR "TGF" OR "adrenomedullin" OR "arteriogenes*" OR "vasculogenes*" OR "vasculo-genes*")	

Cochrane Library Session Results (16 Jun 2022)

#3	**#1 AND #2**	31
#2	(myoma* OR leiomyoma* OR fibroid* OR fibroma* OR fibromyoma* OR angiomyoma* OR angioleiomyoma*):ab,ti,kw	2,547
#1	(pericyt* OR (vascular NEXT endothel*) OR (capillary NEXT endothel*) OR angioprotein* OR angiogenesis OR antiangiogenesis OR neoangiogenesis OR angiogenetic OR antiangiogenetic OR angiogenic OR antiangiogenic OR angiogenin OR angiostatic OR neovasculari* OR (neo NEXT vasculari*) OR angiopoietin* OR (angio NEXT poietin*) OR angiostatin* OR (angio NEXT statin*) OR endostatin* OR vegf* OR (Vascular NEXT Endothelial NEXT Growth NEXT Factor*) OR Vasculotropin OR (c NEXT fos NEXT Induced NEXT Growth NEXT Factor*) OR figf OR (hif NEXT 1*) OR hif1* OR (microvessel NEXT density) OR (micro NEXT vessel NEXT density) OR MVD OR (bloodvessel NEXT density) OR (blood NEXT vessel NEXT density) OR BVD OR (vessel NEXT fragility) OR (vascular NEXT fragility) OR (vessel NEXT wall*) OR VSMC OR (vascular NEXT smooth NEXT muscle NEXT cell*) OR (HB NEXT EGF) OR FGF OR FGFR* OR (fibroblast NEXT growth NEXT factor) OR PDGF OR (platelet NEXT derived NEXT growth NEXT factor) OR (Transforming NEXT Growth NEXT Factor*) OR TGF OR adrenomedullin OR arteriogenes* OR vasculogenes* OR (vasculo NEXT genes*)):ab,ti,kw	20,482

Search 2: Role of angiogenesis in endometrium of patients with uterine fibroids and

1. Fibroids
2. Angiogenesis
3. Infertility

PubMed Session Results (16 Jun 2022)

#4	**#1 AND #2 AND #3**	55
#3	"Fertility"[Mesh] OR "Infertility, Female"[Mesh] OR fertilit*[tiab] OR infertilit*[tiab] OR subfertilit*[tiab] OR nidation*[tiab] OR prenidation*[tiab] OR postnidation*[tiab] OR fecundit*[tiab] OR "Abortion, Spontaneous"[Mesh:NoExp] OR "Abortion, Missed"[Mesh] OR abortion*[tiab] OR miscarr*[tiab] OR "embryo loss*"[tiab] OR "pregnancy loss*"[tiab]	472,451

	OR "Embryonic Development"[Mesh] OR Embryogenes*[tiab] OR "Embryo-genes*"[tiab] OR ((embryo*[tiab]) AND (development[tiab] OR induction[tiab] OR implantation*[tiab]))	
#2	"Myoma"[Mesh:NoExp] OR "Leiomyoma"[Mesh] OR "myoma*"[tiab] OR "leiomyoma*"[tiab] OR "fibroid*"[tiab] OR "fibroma*"[tiab] OR "fibromyoma*"[tiab] OR "angiomyoma*"[tiab] OR "angioleiomyoma*"[tiab]	45,966
#1	"Endothelium, Vascular"[Mesh] OR "Microvessels"[Mesh:NoExp] OR "Capillaries"[Mesh] OR "Pericytes"[Mesh] OR "Angiogenic Proteins"[Mesh] OR "Angiogenesis Modulating Agents"[Mesh] OR "Neovascularization, Physiologic"[Mesh] OR "Neovascularization, Pathologic"[Mesh] OR "Receptors, Fibroblast Growth Factor"[Mesh:NoExp] OR "Receptor, Fibroblast Growth Factor, Type 1"[Mesh] OR "Receptor, Fibroblast Growth Factor, Type 2"[Mesh] OR "Receptor, Fibroblast Growth Factor, Type 4"[Mesh] OR "Fibroblast Growth Factors"[Mesh] OR "Platelet-Derived Growth Factor"[Mesh] OR "Transforming Growth Factor alpha"[Mesh] OR "Transforming Growth Factor beta"[Mesh] OR "Adrenomedullin"[Mesh] OR "pericyt*"[tiab] OR "vascular endothel*"[tiab] OR "capillary endothel*"[tiab] OR "angioprotein*"[tiab] OR "angiogenesis"[tiab] OR "antiangiogenesis"[tiab] OR "neoangiogenesis"[tiab] OR "angiogenetic"[tiab] OR "antiangiogenetic"[tiab] OR "angiogenic"[tiab] OR "antiangiogenic"[tiab] OR "angiogenin"[tiab] OR "angiostatic"[tiab] OR "neovasculari*"[tiab] OR "neo-vasculari*"[tiab] OR "angiopoietin*"[tiab] OR "angio-poietin*"[tiab] OR "angiostatin*"[tiab] OR "angio-statin*"[tiab] OR "endostatin*"[tiab] OR "vegf*"[tiab] OR "Vascular Endothelial Growth Factor*"[tiab] OR "Vasculotropin"[tiab] OR "c-fos-Induced Growth Factor*"[tiab] OR "figf"[tiab] OR "hif-1*"[tiab] OR "hif1*"[tiab] OR "microvessel density"[tiab] OR "micro vessel density"[tiab] OR "MVD"[tiab] OR "bloodvessel density"[tiab] OR "blood vessel density"[tiab] OR "BVD"[tiab] OR "vessel fragility"[tiab] OR "vascular fragility"[tiab] OR "vessel wall*"[tiab] OR "VSMC"[tiab] OR "vascular smooth muscle cell*"[tiab] OR "HB-EGF"[tiab] OR "FGF"[tiab] OR "FGFR*"[tiab] OR "fibroblast growth factor"[tiab] OR "PDGF"[tiab] OR "platelet derived growth factor"[tiab] OR "Transforming Growth Factor*"[tiab] OR "TGF"[tiab] OR "adrenomedullin"[tiab] OR "arteriogenes*"[tiab] OR "vasculogenes*"[tiab] OR "vasculo-genes*"[tiab]	609,829

Embase.com Session Results (16 Jun 2022)

#4	**#1 AND #2 AND #3**	215
#3	'infertility'/de OR 'female infertility'/exp OR 'subfertility'/exp OR 'spontaneous abortion'/exp OR 'missed abortion'/exp OR 'embryo development'/exp OR fertilit*:ab,ti,kw OR infertilit*:ab,ti,kw OR subfertilit*:ab,ti,kw OR nidation*:ab,ti,kw OR prenidation*:ab,ti,kw OR postnidation*:ab,ti,kw OR fecundit*:ab,ti,kw OR abortion*:ab,ti,kw OR miscarr*:ab,ti,kw OR 'embryo loss*':ab,ti,kw OR 'pregnancy loss*':ab,ti,kw OR embryogenes*:ab,ti,kw OR 'embryo-genes*':ab,ti,kw OR ((embryo*:ab,ti,kw) AND (development:ab,ti,kw OR induction:ab,ti,kw OR implantation*:ab,ti,kw))	645,427

#2	'myoma'/de OR 'leiomyoma'/exp OR 'uterus myoma'/exp OR myoma*:ab,ti,kw OR leiomyoma*:ab,ti,kw OR fibroid*:ab,ti,kw OR fibroma*:ab,ti,kw OR fibromyoma*:ab,ti,kw OR angiomyoma*:ab,ti,kw OR angioleiomyoma*:ab,ti,kw	66,869
#1	'vascular endothelium'/exp OR 'microvasculature'/de OR 'capillary'/de OR 'capillary endothelium'/de OR 'pericyte'/de OR 'angiogenesis'/exp OR 'neovascularization (pathology)'/de OR 'fibroblast growth factor receptor'/exp OR 'fibroblast growth factor receptor 1'/exp OR 'fibroblast growth factor receptor 2'/exp OR 'fibroblast growth factor receptor 4'/exp OR 'adrenomedullin'/exp OR 'angiogenesis modulator'/exp OR 'angiogenic protein'/exp OR 'angiogenin'/exp OR 'angiopoietin'/exp OR 'angiopoietin 1'/exp OR 'angiopoietin 2'/exp OR 'endothelial cell growth factor'/exp OR 'fibroblast growth factor'/exp OR 'fibroblast growth factor 1'/exp OR 'fibroblast growth factor 10'/exp OR 'fibroblast growth factor 2'/exp OR 'platelet derived growth factor'/exp OR 'platelet derived growth factor A'/exp OR 'platelet derived growth factor AA'/exp OR 'platelet derived growth factor AB'/exp OR 'platelet derived growth factor B'/exp OR 'platelet derived growth factor BB'/exp OR 'platelet derived growth factor C'/exp OR 'platelet derived growth factor D'/exp OR 'transforming growth factor alpha'/exp OR 'transforming growth factor beta'/exp OR 'vasculotropin'/exp OR 'vasculotropin 121'/exp OR 'vasculotropin 165'/exp OR 'vasculotropin A'/exp OR 'vasculotropin B'/exp OR pericyt*:ab,ti,kw OR 'vascular endothel*':ab,ti,kw OR 'capillary endothel*':ab,ti,kw OR angioprotein*:ab,ti,kw OR angiogenesis:ab,ti,kw OR antiangiogenesis:ab,ti,kw OR neoangiogenesis:ab,ti,kw OR angiogenetic:ab,ti,kw OR antiangiogenetic:ab,ti,kw OR angiogenic:ab,ti,kw OR antiangiogenic:ab,ti,kw OR angiogenin:ab,ti,kw OR angiostatic:ab,ti,kw OR neovasculari*:ab,ti,kw OR 'neo-vasculari*':ab,ti,kw OR angiopoietin*:ab,ti,kw OR 'angio-poietin*':ab,ti,kw OR angiostatin*:ab,ti,kw OR 'angio-statin*':ab,ti,kw OR endostatin*:ab,ti,kw OR vegf*:ab,ti,kw OR 'Vascular Endothelial Growth Factor*':ab,ti,kw OR Vasculotropin:ab,ti,kw OR 'c-fos-Induced Growth Factor*':ab,ti,kw OR figf:ab,ti,kw OR 'hif-1*':ab,ti,kw OR hif1*:ab,ti,kw OR 'microvessel density':ab,ti,kw OR 'micro vessel density':ab,ti,kw OR MVD:ab,ti,kw OR 'bloodvessel density':ab,ti,kw OR 'blood vessel density':ab,ti,kw OR BVD:ab,ti,kw OR 'vessel fragility':ab,ti,kw OR 'vascular fragility':ab,ti,kw OR 'vessel wall*':ab,ti,kw OR VSMC:ab,ti,kw OR 'vascular smooth muscle cell*':ab,ti,kw OR 'HB-EGF':ab,ti,kw OR FGF:ab,ti,kw OR FGFR*:ab,ti,kw OR 'fibroblast growth factor':ab,ti,kw OR PDGF:ab,ti,kw OR 'platelet derived growth factor':ab,ti,kw OR 'Transforming Growth Factor*':ab,ti,kw OR TGF:ab,ti,kw OR adrenomedullin:ab,ti,kw OR arteriogenes*:ab,ti,kw OR vasculogenes*:ab,ti,kw OR 'vasculo-genes*':ab,ti,kw	1,183,130

Web of Science Session Results (16 Jun 2022)

#4	**#1 AND #2 AND #3**	71
#3	TS=("fertilit*" OR "infertilit*" OR "subfertilit*" OR "nidation*" OR "prenidation*" OR "postnidation*" OR "fecundit*" OR "abortion*" OR "miscarr*" OR "embryo loss*" OR "pregnancy loss*" OR "embryogenes*" OR "embryo-genes*" OR (("embryo*") AND ("development" OR "induction" OR "implantation*")))	533,843

#2	TS=("myoma*" OR "leiomyoma*" OR "fibroid*" OR "fibroma*" OR "fibromyoma*" OR "angiomyoma*" OR "angioleiomyoma*")	38,134
#1	TS=("pericyt*" OR "vascular endothel*" OR "capillary endothel*" OR "angioprotein*" OR "angiogenesis" OR "antiangiogenesis" OR "neoangiogenesis" OR "angiogenetic" OR "antiangiogenetic" OR "angiogenic" OR "antiangiogenic" OR "angiogenin" OR "angiostatic" OR "neovasculari*" OR "neo-vasculari*" OR "angiopoietin*" OR "angio-poietin*" OR "angiostatin*" OR "angio-statin*" OR "endostatin*" OR "vegf*" OR "Vascular Endothelial Growth Factor*" OR "Vasculotropin" OR "c-fos-Induced Growth Factor*" OR "figf" OR "hif-1*" OR "hif1*" OR "microvessel density" OR "micro vessel density" OR "MVD" OR "bloodvessel density" OR "blood vessel density" OR "BVD" OR "vessel fragility" OR "vascular fragility" OR "vessel wall*" OR "VSMC" OR "vascular smooth muscle cell*" OR "HB-EGF" OR "FGF" OR "FGFR*" OR "fibroblast growth factor" OR "PDGF" OR "platelet derived growth factor" OR "Transforming Growth Factor*" OR "TGF" OR "adrenomedullin" OR "arteriogenes*" OR "vasculogenes*" OR "vasculo-genes*")	608,677

Cochrane Library Session Results (16 Jun 2022)

#4	**#1 AND #2 AND #3**	4
#3	(fertilit* OR infertilit* OR subfertilit* OR nidation* OR prenidation* OR postnidation* OR fecundit* OR abortion* OR miscarr* OR (embryo NEXT loss*) OR (pregnancy NEXT loss*) OR embryogenes* OR (embryo NEXT genes*) OR ((embryo*) AND (development OR induction OR implantation*))):ab,ti,kw	20,245
#2	(myoma* OR leiomyoma* OR fibroid* OR fibroma* OR fibromyoma* OR angiomyoma* OR angioleiomyoma*):ab,ti,kw	2,547
#1	(pericyt* OR (vascular NEXT endothel*) OR (capillary NEXT endothel*) OR angioprotein* OR angiogenesis OR antiangiogenesis OR neoangiogenesis OR angiogenetic OR antiangiogenetic OR angiogenic OR antiangiogenic OR angiogenin OR angiostatic OR neovasculari* OR (neo NEXT vasculari*) OR angiopoietin* OR (angio NEXT poietin*) OR angiostatin* OR (angio NEXT statin*) OR endostatin* OR vegf* OR (Vascular NEXT Endothelial NEXT Growth NEXT Factor*) OR Vasculotropin OR (c NEXT fos NEXT Induced NEXT Growth NEXT Factor*) OR figf OR (hif NEXT 1*) OR hif1* OR (microvessel NEXT density) OR (micro NEXT vessel NEXT density) OR MVD OR (bloodvessel NEXT density) OR (blood NEXT vessel NEXT density) OR BVD OR (vessel NEXT fragility) OR (vascular NEXT fragility) OR (vessel NEXT wall*) OR VSMC OR (vascular NEXT smooth NEXT muscle NEXT cell*) OR (HB NEXT EGF) OR FGF OR FGFR* OR (fibroblast NEXT growth NEXT factor) OR PDGF OR (platelet NEXT derived NEXT growth NEXT factor) OR (Transforming NEXT Growth NEXT Factor*) OR TGF OR adrenomedullin OR arteriogenes* OR vasculogenes* OR (vasculo NEXT genes*)):ab,ti,kw	20,482

Supplementary file S2 – Modified Newcastle-Ottawa quality assessment

CASE CONTROL STUDIES

Note: A study can be awarded a maximum of one star for each numbered item within the Selection and Exposure categories. A maximum of two stars can be given for Comparability.

Selection

1. Is the case definition adequate?
    a. Yes, with independent validation ☆
    b. Yes, e.g. record linkage or based on self-reports
    c. No description
2. Representativeness of the cases
    a. Consecutive or obviously representative series of cases ☆
    b. Potential for selection biases or not stated
3. Selection of Controls
    a. Community controls ☆
    b. Hospital controls
    c. No description
4. Definition of Controls
    a. No history of disease (search 1: uterine *fibroids and AUB, search 2: uterine fibroids*)
    b. No description of source ☆

Comparability

1. Comparability of cases and controls on the basis of the design or analysis
    a. Study controls for age, defined as reproductive age (15-49 years) according to WHO-guidelines[46] ☆
    b. Study controls for any additional factor: groups matching for history of hormonal use yes/no ☆

Exposure

1. Ascertainment of exposure
    a. Secure record (e.g. surgical records) ☆
    b. Structured interview where blind to case/control status ☆
    c. Interview not blinded to case/control status
    d. Written self-report or medical record only
    e. No description
2. Same method of ascertainment for cases and controls
    a. Yes ☆
    b. No
3. Non-Response rate
    a. Same rate for both groups ☆
    b. Non respondents described
    c. Rate different and no designation

COHORT STUDIES

Note: A study can be awarded a maximum of one star for each numbered item within the Selection and Outcome categories. A maximum of two stars can be given for Comparability

Selection

1. Representativeness of the exposed cohort
   a. Truly representative of the average *women with uterine fibroids and with or without AUB* in the community ☆
   b. Somewhat representative of the average *women with AUB and/or uterine fibroids* in the community.☆
   c. Selected group of users e.g. nurses, volunteers
   d. No description of the derivation of the cohort
2. Selection of the non-exposed cohort
   a. Drawn from the same community as the exposed cohort ☆
   b. Drawn from a different source
   c. No description of the derivation of the non-exposed cohort
3. Ascertainment of exposure
   a. Secure record (e.g. surgical records) ☆
   b. Structured interview☆
   c. Written self-report
   d. No description
4. Demonstration that outcome of interest was not present at start of study
   a. Yes ☆
   b. No

Comparability

1. Comparability of cohorts on the basis of the design or analysis
   a. Study controls for age, defined as reproductive age (15-49 years) according to WHO-guidelines.[46] ☆
   b. Study controls for any additional factor**:** groups matching for history of hormonal use yes/no ☆

Outcome

1. Assessment of outcome
   a. Independent blind assessment ☆
   b. Record linkage☆
   c. Self-report
   d. No description
2. Was follow-up long enough for outcomes to occur
   a. Yes (Minimal 1 month) ☆
   b. No
3. Adequacy of follow-up of cohorts
   a. Complete follow-up: all subjects accounted for ☆
   b. Subjects lost to follow up unlikely to introduce bias - small number lost: *defined as 10%* lost to follow-up. ☆
   c. Follow-up rate < 90% and no description of those lost
   d. No statement

Thresholds for converting the Newcastle-Ottawa scales to Agency for Healthcare Research and Quality (AHRQ) standards (good, fair, and poor):

- **Good quality:** 3 or 4 stars in selection domain AND 1 or 2 stars in comparability domain AND 2 or 3 stars in outcome/exposure domain
- **Fair quality:** 2 stars in selection domain AND 1 or 2 stars in comparability domain AND 2 or 3 stars in outcome/exposure domain
- **Poor quality:** 0 or 1 star in selection domain OR 0 stars in comparability domain OR 0 or 1 stars in outcome/exposure domain

Risk of Bias assessment of the non-randomized trials according to Newcastle-Ottawa Scale[45].

		Selection				Comparability		Outcome			Total ★	Overall quality
**Author**	**Year**	**1**	**2**	**3**	**4**	**1A Age**	**1B Hormone use**	**1**	**2**	**3**		**AHRQ Standards**
**Cohort studies search 1: Uterine fibroids, AUB and pharmaceutical therapy**												
Maia	2008	A★	A★	A★	A★	Yes★	No	A★	B	A★	8	GOOD
**Case-control studies search 1: Uterine fibroids, AUB and pharmaceutical therapy**												
Anania	1997	A★	A★	A★	A★	Yes★	Yes★	A★	A★	A★	9	GOOD
Bereza	2014	A★	B	C	B	No	No	A★	A★	C	4	POOR
Governini	2021	A★	A★	B	A★	Yes★	Yes★	A★	A★	A★	8	GOOD
Hague	2000	B	B	B	A★	Yes★	Yes★	A★	A★	C	5	POOR
Khan	2010a	A★	A★	B	B	Yes★	Yes★	A★	A★	C	6	FAIR
Khan	2010b	A★	A★	B	B	Yes★	Yes★	A★	A★	C	6	FAIR
Kolanska	2019	B	B	C	B	Yes★	Yes★	A★	A★	C	4	POOR
Makhija	2008	B	B	A★	A★	No	No	A★	A★	C	4	POOR
Oh	2013	A★	B	B	A★	Yes★	Yes★	A★	A★	C	6	FAIR
Zhang	2010b	A★	A★	A★	A★	Yes★	Yes★	A★	A★	A★	9	GOOD
**Case-control studies search 2: Uterine fibroids and infertility**												
Doherty	2015	A★	A★	A★	A★	Yes★	No	A★	A★	C	7	FAIR
Kozachenko	2020	C	B	B	A★	Yes★	No	A★	A★	A★	5	POOR
Novin	2018	A★	A★	A★	A★	Yes★	Yes★	A★	A★	A★	9	GOOD
Sinclair	2011	A★	A★	A★	A★	No	No	A★	A★	A★	7	FAIR
